# Modelling brain development to detect white matter injury in term and preterm born neonates

**DOI:** 10.1093/brain/awz412

**Published:** 2020-01-16

**Authors:** Jonathan O'Muircheartaigh, Emma C Robinson, Maximillian Pietsch, Thomas Wolfers, Paul Aljabar, Lucilio Cordero Grande, Rui P A G Teixeira, Jelena Bozek, Andreas Schuh, Antonios Makropoulos, Dafnis Batalle, Jana Hutter, Katy Vecchiato, Johannes K Steinweg, Sean Fitzgibbon, Emer Hughes, Anthony N Price, Andre Marquand, Daniel Reuckert, Mary Rutherford, Joseph V Hajnal, Serena J Counsell, A David Edwards

**Affiliations:** 1 Department of Forensic and Neurodevelopmental Sciences, Sackler Institute for Translational Neurodevelopment, Institute of Psychiatry, Psychology and Neuroscience, King’s College London, London, UK; 2 Centre for the Developing Brain, School Biomedical Engineering and Imaging Sciences, King’s College London, St Thomas’ Hospital, London, UK; 3 MRC Centre for Neurodevelopmental Disorders, King’s College London, London, UK; 4 Department of Bioengineering, Imperial College London, London, UK; 5 Donders Centre for Cognitive Neuroimaging, Donders Institute for Brain, Cognition and Behaviour, Radboud University, Nijmegen, The Netherlands; 6 Department of Cognitive Neuroscience, Radboud University Medical Centre, Nijmegen, The Netherlands; 7 Faculty of Electrical Engineering and Computing, University of Zagreb, Zagreb, Croatia; 8 Biomedical Image Analysis Group, Department of Computing, Imperial College London, London, UK; 9 Centre for Functional MRI of the Brain (FMRIB), Wellcome Centre for Integrative Neuroimaging, Nuffield Department of Clinical Neurosciences, University of Oxford, Oxford, UK; 10 Department of Neuroimaging, Centre for Neuroimaging Sciences, Institute of Psychiatry, King’s College London, London, UK

**Keywords:** neonatology, imaging methodology, brain development, neuroanatomy, neuropathology

## Abstract

Premature birth occurs during a period of rapid brain growth. In this context, interpreting clinical neuroimaging can be complicated by the typical changes in brain contrast, size and gyrification occurring in the background to any pathology. To model and describe this evolving background in brain shape and contrast, we used a Bayesian regression technique, Gaussian process regression, adapted to multiple correlated outputs. Using MRI, we simultaneously estimated brain tissue intensity on T_1_- and T_2_-weighted scans as well as local tissue shape in a large cohort of 408 neonates scanned cross-sectionally across the perinatal period. The resulting model provided a continuous estimate of brain shape and intensity, appropriate to age at scan, degree of prematurity and sex. Next, we investigated the clinical utility of this model to detect focal white matter injury. In individual neonates, we calculated deviations of a neonate’s observed MRI from that predicted by the model to detect punctate white matter lesions with very good accuracy (area under the curve > 0.95). To investigate longitudinal consistency of the model, we calculated model deviations in 46 neonates who were scanned on a second occasion. These infants’ voxelwise deviations from the model could be used to identify them from the other 408 images in 83% (T_2_-weighted) and 76% (T_1_-weighted) of cases, indicating an anatomical fingerprint. Our approach provides accurate estimates of non-linear changes in brain tissue intensity and shape with clear potential for radiological use.

## Teaser

Interpreting neonatal clinical neuroimaging is challenging due to rapid brain maturation over short periods. O'Muircheartaigh *et al.* use statistical modelling to build growth curves of tissue intensity and shape, and show that focal white matter injuries present as deviations from these curves in individual neonates.

See Duerden and Thompson (doi:10.1093/brain/awz421) for a scientific commentary on this article.

## Introduction

Neuroimaging during the perinatal period is both practically and technically challenging (Lodygensky and Thompson, 2017). Over a very short period, the brain changes in size and shape, tissue contrast changes, and transient developmental structures disappear (Kostović and Jovanov-Milošević, 2006). These changes occur rapidly over periods from days to weeks and often follow non-linear and regionally specific trajectories (Dubois *et al.*, 2016; Makropoulos *et al.*, 2016) reflecting regionally and temporally asynchronous developmental processes such as myelination and synaptic proliferation (Harris *et al.*, 2011; Lebenberg *et al.*, 2019). When investigating perinatal brain injury this evolving background represents a substantial hurdle, as imaging changes themselves can be both spatially and temporally heterogeneous (Rutherford *et al.*, 2006).

Because of this complexity, studies have shown inter- and intra-rater reliability in interpreting neonatal MRI to be moderate to low (Morel *et al.*, 2016). This is especially the case with age-related image intensity or shape changes that may indicate dysmaturation, such as diffuse white matter injury, small punctate white matter lesions or ventricular dilation. Sometimes visually subtle, these features may be ‘normal’ or expected in one context but not in another. For example, diffuse white matter high signal intensity may be a general feature of the premature brain, seen in the majority of preterm infants at term age, but it has limited prognostic significance (de Bruïne *et al.*, 2011). Myelination may be disrupted by brain injury and prematurity and the degree of disruption can be dependent on both the actual pathology and the age of insult (Volpe, 2009).

The question for interpreting a clinical neonatal MRI is therefore complex: what is abnormal in the brain, given a particular age and clinical history? Research studies investigating the perinatal period have helped address some of this complexity, creating maps of a typical brain at different gestational ages. Statistical models of brain growth or image intensity change have begun describing development continuously in a way analogous to growth charts (Kuklisova-Murgasova *et al.*, 2011; Dean *et al.*, 2014; Holland *et al.*, 2014). These growth curves often rely on strong assumptions on the shape of that curve that have to be tailored and optimized to different magnetic resonance modalities, brain regions and age spans (Mills and Tamnes, 2014). This has led to clinical studies being restricted to narrow or fixed age ranges (Oishi *et al.*, 2019), with increased statistical power but a reduced likelihood of clinical translation.

As an alternative, non-parametric approaches to modelling normative developmental variation have been proposed that are less dependent on strong hypotheses of the shape of a curve and have been successfully applied in neuroimaging data (Ziegler *et al.*, 2014; Marquand *et al.*, 2016). An advantage here is that, just as with standard growth curves, the resulting models can be used to generate a score characterizing the deviation of an individual subject from an expected average shape/intensity (as a percentile or *Z*-score) but with respect to multiple clinically meaningful variables, not just age or sex. Importantly, this can be quantified in single observations of an individual. In the context of prematurity, this property is especially important as brain effects are both clinically and spatially variable so group average comparisons may occlude real effects (Sled and Nossin-Manor, 2013).

In this work, we take advantage of multi-contrast structural MRI data acquired across a wide range of ages as part of the developing Human Connectome Project (dHCP). We use a Bayesian non-parametric model estimation technique, Gaussian process regression (GPR), implemented within a multi-output framework (Álvarez *et al.*, 2011) so as to be able to take advantage of cross-sectional correlation between the outputs (Liu *et al.*, 2018), and therefore provide better predictions. We simultaneously model the mean and expected variance of tissue intensities and shape from anatomical T_1_- and T_2_-weighted images sampled across the perinatal period (26 to 45 weeks) in both premature and term-born neonates. From this we derive a family of multimodal 4D growth curves, providing statistical measures of variation across the cohort. From this we show that individuals develop along trajectories defined by these growth curves and have a multimodal brain imaging fingerprint that persists with increasing chronological age; that focal abnormalities such as punctate white matter lesions are reflected by deviations from these typical trajectories at a voxel level in individual infants, allowing accurate automated detection of lesions; and that the global effects such as premature birth can lead to detectable deviations in global and local brain morphology, which can also be quantified by data-driven approaches.

## Materials and methods

### Participants

**Figure 1:**
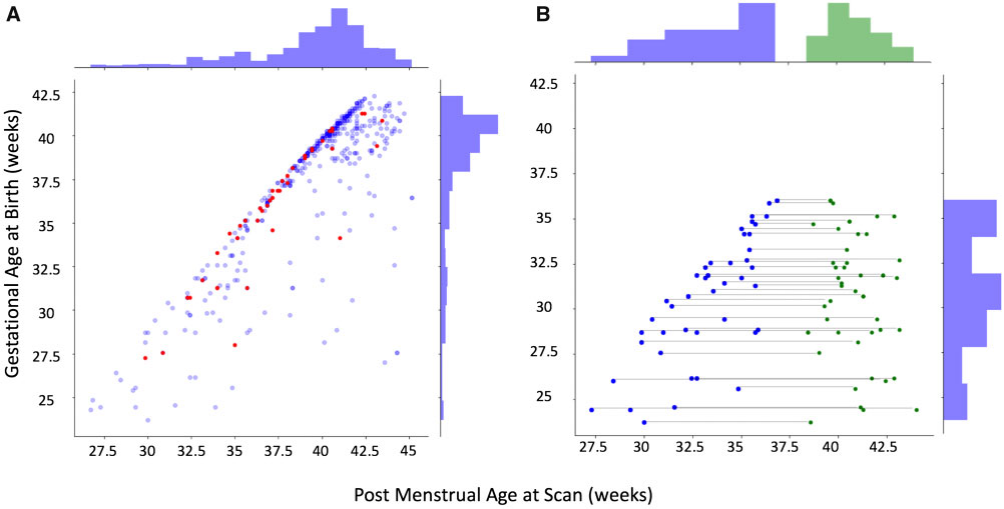
**Sample demographics.** (**A**) Age distribution of the infants that contributed towards model training (blue); infants with punctate white matter lesions (*n *= 40), held out of the training dataset, are highlighted in red. (**B**) In a premature born subset of this larger cohort (*n *= 46), additional repeat scans (green dots) were available at term equivalent age. Repeat scans were also held out of the training set.

All subjects participated in the dHCP, and written informed parental consent was obtained before enrolment to the study. The training sample consisted of 408 (189 female) neonates ranging in age at scan (post-menstrual age; PMA) from 26 to 44 weeks, with a gestational age at birth (GA) of 23–42 weeks (mean PMA 39.2 weeks, mean GA 37.4 weeks) (Fig. 1A). Datasets acquired from both singletons and twins were included in this study. These images were visually inspected and datasets with substantial motion on MRI or major focal parenchymal lesions at the time of their first scan were excluded. We did not exclude neonates with radiologically-reported punctate white matter lesions (PWML), small subependymal cysts or small haemorrhages in the caudothalamic notch, as these are a common finding in the preterm population in particular.

Two further datasets were included. Of the 408 neonates in the training sample, 46 of those born preterm (mean PMA at first scan 33.5 weeks, range 27–36, mean GA 30.9 weeks, range 23–36 weeks) had an additional scan on a second occasion (mean PMA at second scan 40.7 weeks, range 38–44 weeks) (Fig. 1B). In addition, an independent dataset of 40 neonates with PWML were identified. See Table 1 and Fig. 1 for summary sample demographics and sample age distribution.

**Table 1:** Sample demographics

	Training	Longitudinal		**PWML** ^b^	Template
		**Time 1** ^a^	**Time 2** ^b^
Sample size	408	46	–	40	20
Postmenstrual age at scan, weeks					
Mean	39.3	33.5	40.8	37.2	37.4
Median	40.3	34.3	40.7	37.1	38.4
Range	26–45	28–37	38–43	29–43	29–42
Gestational age at birth, weeks					
Mean	37.6	30.9	–	35.9	34.6
Median	39.14	31.3	–	36.3	36
Range	23–42	24–36	–	27–41	23–41
Sex					
Male	219	28	–	23	12
Female	189	18	–	17	8

a This sample is included in the training dataset.

b This sample is held out of the training dataset.

All datasets were acquired on a Philips Achieva 3 T scanner at the Evelina Newborn Imaging Centre using a dedicated 32-channel neonatal head coil (Hughes *et al.*, 2017). All anatomical volumes were collected as part of the dHCP and are described in detail in Makropoulos *et al.* (2018).

For both T_1_- and T_2_-weighted anatomical scans, turbo spin echo (TSE) sequences were used with two stacks per weighting, sagittal and axial. For T_2_-weighted scans, the parameters were: repetition time = 12 s, echo time = 156, SENSE = 2.11 (axial) and SENSE = 2.58 (sagittal). For T_1_-weighted stacks an inversion recovery TSE sequence was used. Images were acquired with a repetition time = 4.8 s, echo time = 8.7, inversion time = 1740, SENSE factor of 2.26 (axial) and 2.66 (sagittal). For all images the in-plane resolution was 0.8 × 0.8 mm with a slice thickness of 1.6 mm, with a slice overlap of 0.8 mm. The resulting images were motion corrected as described in Cordero‐Grande *et al.* (2018) and super-resolution reconstruction was performed as in Kuklisova-Murgasova *et al.* (2012), resulting in 3D volumes resampled to 0.5 mm isotropic resolution, taking between 30 min and 3 h on a GPU (graphics processing unit) depending on the input data size (number of slices). The resulting images were also corrected for bias field inhomogeneities.

### Initial image registration to a common space

Twenty individual neonatal datasets were selected across a wide age range from 29 to 43 weeks (Table 1). A combined representative template was created from this sample based on two imaging features: T_2_-weighted image volume intensity and the cortical mantle (the tissue between the white/grey matter boundary matter and pial surface), derived from the dHCP structural pipeline (Makropoulos *et al.*, 2018). This template was created using the antsMultivariateTemplateConstruction.sh script (Avants *et al.*, 2011, https://github.com/ANTsX/ANTs). As contrast and shape changes are rapid over this age range, this template image is not representative of any specific age group, but acts as an initial middle space. The sample included in this study is predominantly term age (see histogram in Fig. 1), therefore, the purpose of this template was to be a midpoint in the sample age range, but not representative of the entire sample itself.

All 408 training images, the 46-s scan images and the 40 images with PWML were registered to this common space, with a single MRI modality, the T_2_-weighted image, used for rigid and affine linear registration steps, and two channels, the T_2_-weighted and cortical mantle images, used as input channels for the non-linear registration step. The T_1_- and T_2_-weighted images were resampled into this standard space in a single step using BSpline interpolation and the affine and non-linear transformations were recorded. With the exception of this interpolation step performed during resampling, no further spatial smoothing was performed. See Supplementary Fig. 1 for a diagram of the registration process.

### Multiple output Gaussian process regression

To fit the observed intensity and shape data, we used a non-parametric approach, GPR (Rasmussen and Williams, 2006). GPR is a data-driven approach that provides a posterior distribution of functions (here growth curves), given an input dataset and model. The T_1_- and T_2_-weighted images in template space, as well as the inverse displacement fields (the *x*, *y* and *z* component images, describing local tissue shape) were used as the five outputs of a multi-output Gaussian process regression (MOGP). Instead of running five serial models for intensity and shape, we used this MOGP to capitalize on the shared information between our model outputs (image intensity and shape, which occur concurrently over this age period). The used multi-output model was the intrinsic co-regionalization model as summarized in Álvarez *et al.* (2012). All Gaussian process model estimations were calculated using the GPy package (https://github.com/SheffieldML/GPy).

The design matrix for the multi-output model coded for PMA, gestational age and sex. A linear sum of three Gaussian process covariance kernels were used when estimating the relationship between the three input and output variables: a linear, a squared exponential and a white noise kernel (Rasmussen and Williams, 2006). These were chosen because of prior work showing a combination of parametric smooth sigmoidal and slower, effectively linear, terms can provide a better fit over wider age ranges in older age groups (Dean *et al.*, 2014). MOGP models were estimated for every voxel (*n *= 292 449) separately and model hyper-parameters were optimized according to log marginal likelihood.

Model accuracy was quantified using a 5-fold cross-validation approach, with every fifth infant, in sequential order of when they took part in the study, left out of each training fold and accuracy quantified using mean absolute error of the predicted image intensity compared to the observed. To test the fidelity of these individual models to the whole brain tissue contrast, the ratio of predicted T_1_- and T_2_-weighted values to observed value was calculated for the entire brain.

To model global shape changes over this period, the affine transformation was decomposed into stretch and shear components and these six elements were modelled separately in a single output GPR with the same design matrix and cross-validation approach as the voxelwise MOGPs. The rotation and translation components were ignored.

To illustrate the model prediction of brain growth in the *ex utero* neonatal brain, we combined the predicted affine and warps from 20 weeks (earlier than we have observations) to 44 weeks (later), with GA fixed to 2 weeks prior to scan (although after 41 weeks, it was fixed to 40 weeks). To illustrate the model prediction of prematurity on the term age brain, we fixed PMA to 41 weeks with estimated GA in weekly increments from 28 to 40 weeks.

For every neonate, a voxelwise deviation score was calculated for their T_1_ and T_2_ intensity images. This was the difference between the model expected mean value (point prediction) and the observed data from each neonate (calculated on its out-of-fold prediction), scaled by the square root of the predicted variance (e.g. standard deviation). This gave a measure of deviance for each image from the expected image intensity or shape in units of standard deviation (*Z*) (termed normative probability maps in Ziegler *et al.*, 2014).

### Quantifying punctate white matter lesions in neonatal structural images

In 40 neonates, PWMLs were identified by two authors (J.O.M. and S.C.) and manually outlined on their T_1_-weighted image in native (acquired) space using fslview, part of the FSL package (Smith *et al.*, 2004) and saved as binary mask volume files. The resulting PWML labels were resampled from native to template space in a single nearest-neighbour interpolation step. These PWML labels were used as true positive labels.

As punctate lesions can be very small, we avoided performing any smoothing on the T_1_-weighted contrast images, though this carries a risk of a high level of false positives (Salmond *et al.*, 2002). To address this, we used a patch-based anomaly detection method as described by Mah *et al.* (2014). In short, for every voxel in a PWML dataset Z-map we (i) extracted a patch of voxels in that voxel’s immediate neighbourhood (*n *= 27); (ii) calculated the similarity of the extracted patch with the same patch in a reference set of 80 other neonates randomly sampled; and (iii) calculated a ‘zeta’ distance from the individual neonate patch to the nearest of *k*-clusters in the reference set (here *k* was set to 8), providing a type of outlier magnitude index. This index takes into account neighbouring voxels without resorting to blurring the underlying data.

Receiver operator characteristic (ROC) curves were used to quantify the ability of these normative probability maps to classify PWMLs, as in O’Muircheartaigh *et al.* (2019). For the T_1_-weighted scans only, the area under the ROC curve (AUC) was calculated for every neonate using (i) the simple sample *Z*-score; (ii) the MOGP calculated *Z*-score; and (iii) the zeta anomaly score. These AUCs were compared pairwise using a non-parametric Friedman test followed by *post hoc* Wilcoxon test (Demšar, 2006).

### Quantifying longitudinal consistency of deviation maps

In a subset of 46 neonates with a second scan acquired entirely held out from the model construction (Fig. 1B), we quantified their deviation from the predicted T_1_- and T_2_-weighted intensity images and calculated the spatial correlation of this map with the 408 other deviation maps calculated earlier. We ranked the resulting correlations and counted the number of times the most similar image to the second time point was the same neonate at an earlier time point.

### Data availability

The anonymized neuroimaging data that support the findings of this study are available through the developing Human Connectome Project (http://www.developingconnectome.org/project/) in a minimally preprocessed form. Secondary processed neuroimaging datasets (deviation maps and GPR prediction maps) will also be available through this website. The image processing scripts used in the study are available via github https://github.com/jonnyomuir/NeonatalGP.

## Results

**Figure 2:**
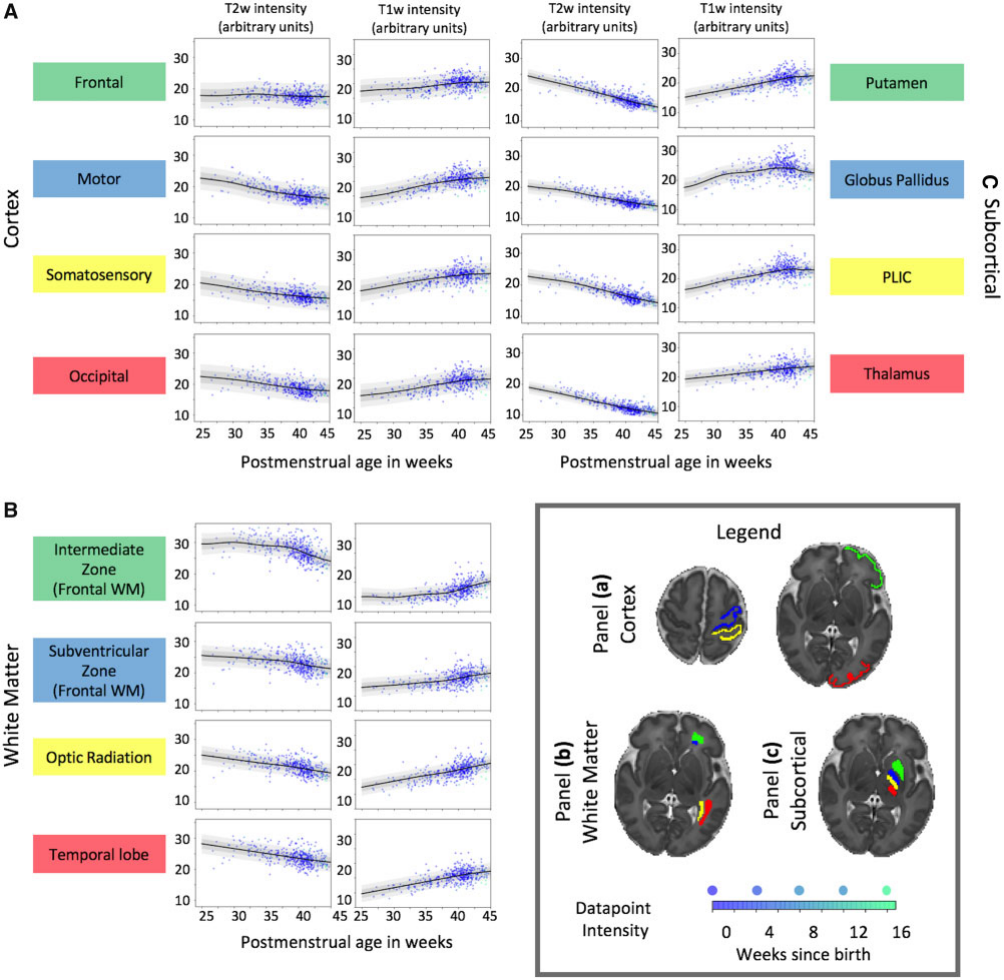
**Image intensity growth charts in regions of interest.** Illustrative intensity plots for T_1_- and T_2_-weighted images from (**A**) cortical, (**B**) white matter and (**C**) subcortical regions. Individual data points are overlaid on the 1 (dark grey) and 2 (lighter grey) standard deviation ranges. Mean predictions and prediction interval plots assume the age at birth to be 1 week prior to age at scan. As the effect of prematurity is not shown here, individual data points are shaded by how far the time of scan is from that neonate’s gestational age at birth (darker blue = closer). The majority of term-age scanned neonates are also born at normal term age, only ∼10% were born >4 weeks prior to their scan. PLIC = posterior limbs of the internal capsule; WM = white matter.

The GPR model estimated T_1_- and T_2_-weighted intensity growth curves at every voxel in the brain, with associated continuous estimates of variability. Figure 2 illustrates these curves averaged in three sets of regions of interest delineated on a single slice: cortex (Fig. 2A), white matter and transient periventricular structures (Fig. 2B), and subcortical regions (Fig. 2C). The curves show estimates of image intensity between 25 and 45 weeks PMA. Data points from individual neonates are overlaid on the curves. There is clear variability of growth curve shape, depending on the structure, and direction, depending on contrast. In regions analogous to the subventricular and intermediate zones, there is a rapid change towards term-equivalent age as the structures resolve to white matter. In other white matter regions, the development is linear.

Scaling the entire raw T_1_- and T_2_-weighted image intensity range to the median of the held-out prediction in these regions of interest, acting as a form of intensity normalization, provided closer matches to the curves and the histograms of model deviations were more readily comparable between subjects (Supplementary Fig. 2).

**Figure 3:**
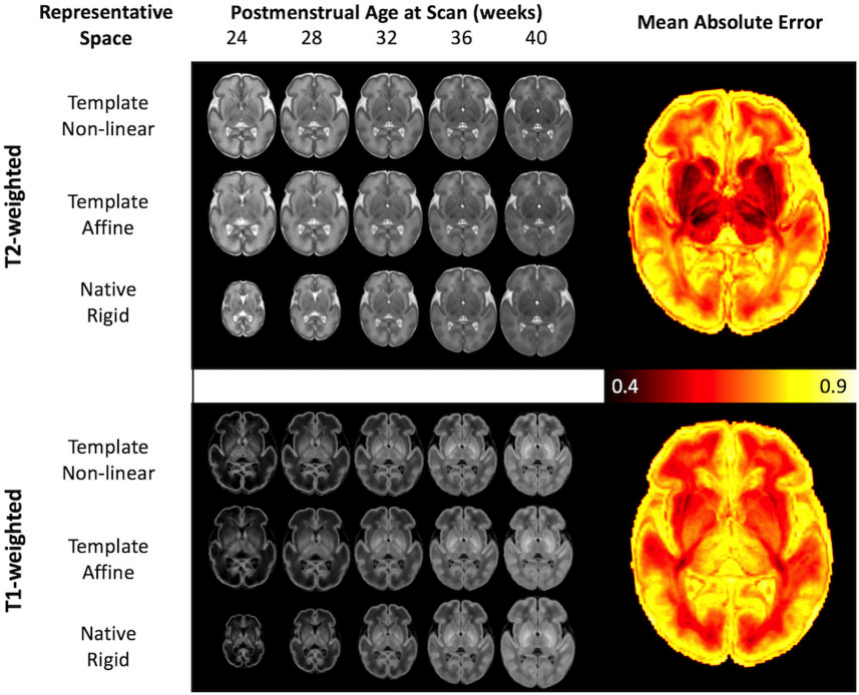
**Model predictions of image shape and intensity over age.** Intensity, shape and native space models visualized for each of T_2_- and T_1_-weighted modalities at fixed PMA, assuming age at birth is 1 week prior to scan. The *top row* for each shows the intensity model in template space (after removing global and local shape changes), the *middle row* after deforming the standard space image back to a representative affine space (only global changes have been removed), and the *bottom row* shows the expected image in native space (where the image represents the expected shape, size and intensity of a neonate at that age. The mean absolute error of the prediction of T_1_ and T_2_-weighted image intensity is shown in the images on the *right*, in units of input data standard deviation. These curves are represented as an animation in Supplementary Videos 1–6.

In addition to image intensity, local tissue shape was estimated using the parameters from the non-linear warps from each individual neonate to the template, and global tissue shape was estimated using the 12 degrees of freedom linear affine parameters to the template. Using these data, Fig. 3 shows a tripartite representation of the T_1_- and T_2_-weighted intensity data: the top row represents intensity changes only (templates have been non-linearly aligned, and thus global and local shape changes have been removed); the middle row shows local shape and intensity changes (templates have only been affinely aligned); and the bottom row shows global, local and intensity changes (templates are only rigidly aligned).

The mean absolute error of the model prediction against the (held-out) raw image intensity is shown on the right of Fig. 3, in units of standard deviation (lower values indicate better performance). The T_2_-weighted images showed the smallest error in subcortical regions and in white matter. The T_1_-weighted images showed the smallest error throughout white matter and cerebellum in particular. Supplementary Videos 1–6 show the GPR estimate of brain development as a function of age at scan, with age at birth fixed at 1 week prior to scan, mirroring the six rows of Fig. 3. Supplementary Videos 7–12 illustrate the estimated effect of prematurity on the brain at a fixed age at scan of 40 weeks and age at birth ranging from 20 to 39 weeks.

### Individual differences from the model are longitudinally consistent towards term age

**Figure 4:**
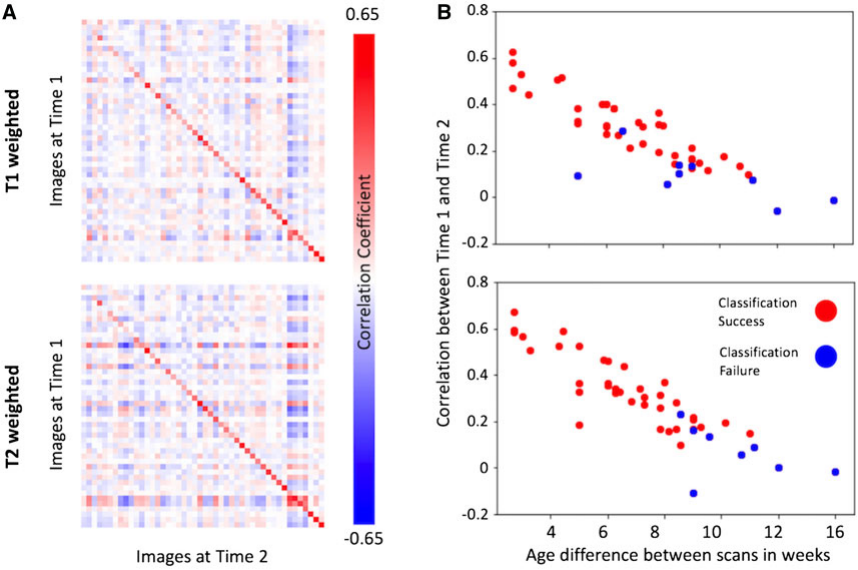
**Longitudinal consistency of model deviations.** In longitudinal data, individual neonates are more similar to themselves in the spatial pattern of their individual differences with respect to the model prediction than other infants (**A**). Pearson’s correlation coefficient matrix between the model deviations of each pair of images (*n *= 46) at Time 1 against all follow-up scans at Time 2, with the images on both axes ordered according to PMA at each scan time. Comparing to the whole cohort of 408 neonates, 35/46 infants are most correlated to themselves at Time 1 on T_1_-weighted images, and 38/46 on T_2_-weighted. The larger the difference in age between scans (**B**), the less similar the images are between two time points from the same individual. Red dots indicate classification successes and blue failures (can Time 1 be identified from Time 2 across all 408 training datasets?).

In 46 neonates who had MRI collected at a second time point, deviations (*z*-scores) from the model predicted T_1_- and T_2_-weighted intensity curves were calculated voxelwise. Comparing these maps to the full cohort of 408 neonates, the deviation maps of these neonates were, on average, more correlated with themselves at Time point 1 (Fig. 4A) than with other neonates. Of the 46-s scans, 35 (72%) were most correlated to their own first time point scan relative to the other 407 scans (T_1_-weighted images), and 38/46 (83%) on T_2_-weighted. However, there was a strong dependence on the time between time points and intrasubject scan similarity (Fig. 4B, r = 0.89, *P *< 0.001).

### Deviations from expected tissue intensity are sensitive to punctate white matter lesions in neonates

**Figure 5:**
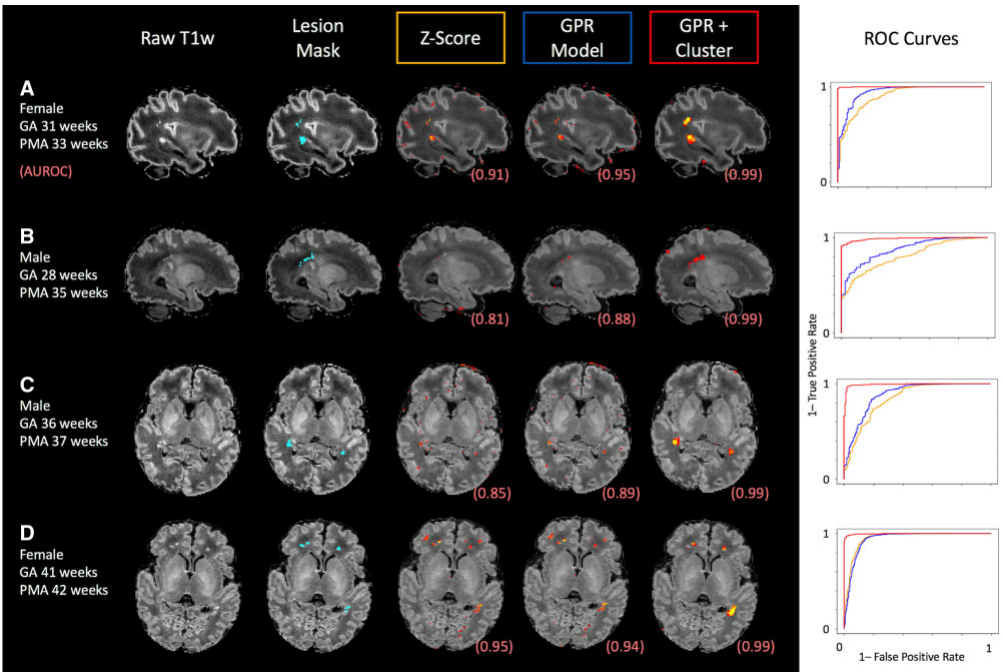
**Four example neonates with punctate white matter lesions.** For each case, the T_1_-weighted image (in template space), the manually delineated PWML masks, and the distribution of the top 0.5% of values for each of the three methods. The ROC curves for each method for each individual infant are also shown on the *right*. GA = gestational age at birth.

In 40 infants with punctate lesions, manually defined masks were outlined and used as positive labels in an ROC analysis. The distribution of these PWMLs is illustrated in Supplementary Fig. 3. The AUC was calculated for each of three methods (a simple sample *Z*-score, deviations from the GPR model, and deviations from the GPR model combined with the zeta outlier detection method) (Mah *et al.*, 2014) and compared using paired *t*-tests. The GPR model significantly outperformed sample *Z*-scores (mean AUC = 0.894 versus 0.865, Wilcoxon W = 98, *P *< 0.001) and cluster enhancement increased specificity, again compared to just the GPR model (mean AUC 0.951 versus 0.894, Wilcoxon W = 57, *P *> 0.0001). Four individual babies are shown in Fig. 5, illustrating punctate lesions and their masks, as well as the first percentile of values of *Z*-scores from the population, from the GPR and after-cluster enhancement. All ROC curves have a single positive class, i.e. we naïvely assumed the only tissue abnormalities for each infant are the outlined PWMLs.

**Figure 6:**
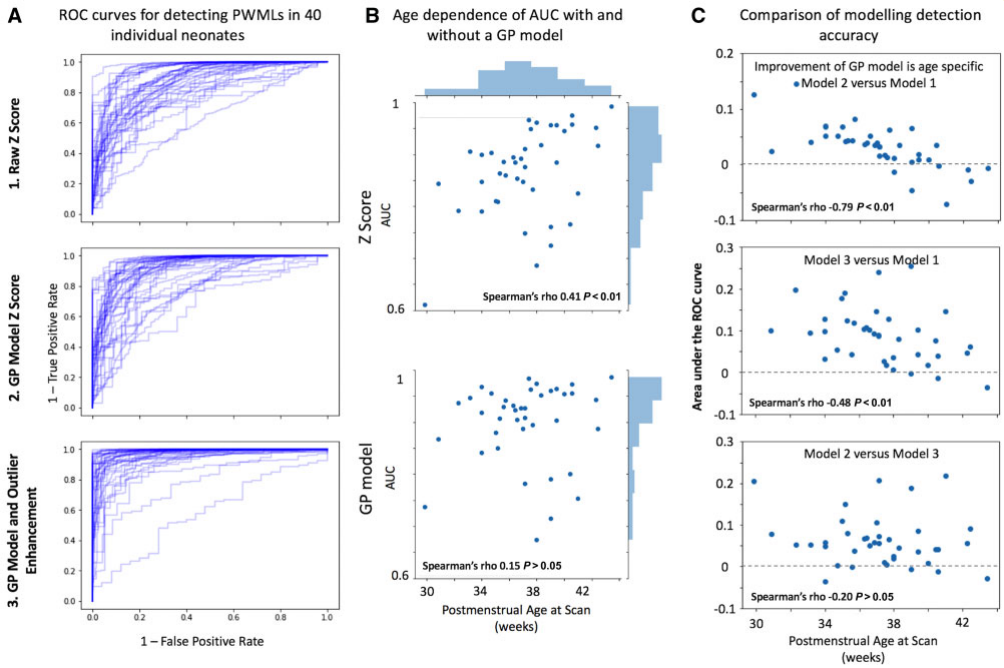
**White matter injury detection performance.** Receiver operating characteristic (ROC) curves for detecting PWMLs in all of the 40 neonates investigated using the three approaches tested here. (**A**) Curves stretched to the top left corner indicated better performance. An age dependence on AUC values is evident without the model (**B**) and reduces substantially when using the Gaussian process (GP) model (**B**). Specifically, PWML detection performance improves in an age-dependent fashion when using a Gaussian process model (**C**, *top*). Outlier detection improves detection performance further (**C**, *middle* and *bottom*).

Figure 6A illustrates the ROC curves for all 40 neonates in individual plots for each of the three methods. Using the raw *Z*-score to quantify PWMLs had an expected dependence between PMA and AUC (Fig. 6B; Spearman rho = 0.41, *P *< 0.01) that was absent using the GPR model. The differences in performance (in terms of AUC) are quantified in Fig. 6C. Using the GPR model provided a mean gain of 0.03 AUC. The performance improvement was significantly associated with age at scan (Fig. 6C; Spearman’s rho = −0.79, *P *< 0.001). The performance boost by using the zeta anomaly detection method over the GPR model alone was not age-dependent (Spearman’s rho = −0.2, *P *> 0.4) but instead was global, with a median increase in AUC of 0.05 for each subject, compared to the GPR model alone (Wilcoxon’s W = 57, *P *< 0.001). The effect of scaling the images to their predicted median value had a consistent positive effect on detection accuracy (in all cases, *P *< 0.001), but the performance gain was quite small, on average AUC improvement <0.01. There was no correlation between PWML spatial size and AUC.

## Discussion

In prematurity, a neonate’s early life experience is very different to that of a typical term-born child with a combination of loss of intrauterine environment, medical treatment, and the hospital environment all providing stressors not experienced by a term-born neonate. Although we are often more interested in why one individual neonate does well or badly, given their own context and circumstances, prior research has tended to focus on group cohort studies, with limited applicability for inferring important differences in a single subject. Using a multi-dimensional approach to model *ex utero* brain development over a wide age window (26–45 weeks PMA), we built continuous charts of brain shape and image intensity, providing a brain-wide abnormality index appropriate to developmental stage and clinical history. In this way, we provide context for growth curves by asking not just what the brain should look like for a given age but also for a given degree of prematurity and sex.

The shape of the resulting intensity curves depended on the structure and content of the tissue being modelled. In areas such as the globus pallidus, which has a contribution from tissue iron, and the early myelinating posterior limb of the internal capsule, the intensity curves from T_1_ and T_2_ signal had subtly different direction and trajectories, reflecting the differential sensitivity and unique information content in these contrasts. Sensory cortex showed more linear tissue intensity changes compared to frontal cortex, which is effectively flat over the perinatal period (Fig. 2A). Developmental changes in global shape were demonstrated in both typical whole brain growth (Supplementary Videos 1 and 4) and local gyrification (Supplementary Videos 2 and 5). The modelled effect of prematurity in the term-born brain replicated observations of mild dolichocephaly in prematurity (Mewes *et al.*, 2007; McCarty *et al.*, 2017) (Supplementary Videos 9 and 12) as well as enlarged ventricles (Peterson *et al.*, 2003) (Supplementary Videos 8 and 11) and lower intensity of myelin signal in the corticospinal tract (Supplementary Videos 7 and 10).

In terms of accuracy of modelling tissue intensity, the model performed very well in white matter as well as cortical areas where morphology is consistent across individual infants and time points, especially the central and temporal sulci and the insula (Fig. 3). Where intersubject sulcal variability is more marked (Li *et al.*, 2015; Dubois *et al.*, 2016; Bozek *et al.*, 2018), blurring/higher error was evident (Fig. 3) especially at later PMA. This apparent blurring is most likely due to the normal inter-individual variability in cortical folding in association cortices seen in adults (Van Essen, 2005) but established just before term age as tertiary folds develop.

This is also likely due to the dependence of this work on volumetric registration. Spatial correspondence between gyri is less optimal than when using a surface representation that can more accurately register cortical areas in particular (Robinson *et al.*, 2014). A model built on a surface representation would also allow a more direct quantification of cortical abnormalities (e.g. thicker cortex, abnormal or immature curvature) that is not possible here but has been useful in other neurodevelopmental applications, such as epilepsy (Adler *et al.*, 2017).

The whole brain deviations of individual neonates are longitudinally consistent. In 83% of neonates with longitudinal data, their pattern of deviation from the GPR intensity model at a second time point identified them uniquely from the full cohort of 408 infants (Fig. 4A), providing an anatomical fingerprint. This consistency was lower when the infant was born at an earlier age (Fig. 4B), around the time of development of secondary gyri (Chi *et al.*, 1977) and while transient structures such as the subplate are still quite prominent (Kostović *et al.*, 2019). Individual differences in these specific areas may simply be incomparable between these developmental stages. An increase in signal prior to the onset of myelination may be a decrease in the same area later on depending on the underlying developmental processes then occurring (e.g. myelination) or the underlying pathology/injury (inflammation versus bleed).

From a practical study design and interpretation perspective, this indicates that individual differences measured at very early PMA could have different associations with any form of outcome measure at older ages compared to individual differences at later PMA. Previous studies in infants and older toddlers have demonstrated an age-dependence on detecting brain-behaviour associations, with consistent relationships between anatomy (in this case myelin, a predominantly postnatal process) and cognitive ability being apparent from around 2 years of age as gross brain growth slows (O’Muircheartaigh *et al.*, 2013; Dean *et al.*, 2014). The important question to address in the future will be when this longitudinal consistency stabilizes (e.g. in longitudinal studies that cover wider age ranges, for example in the Baby Connectome Project) (Howell *et al.*, 2019).

**Figure 7:**
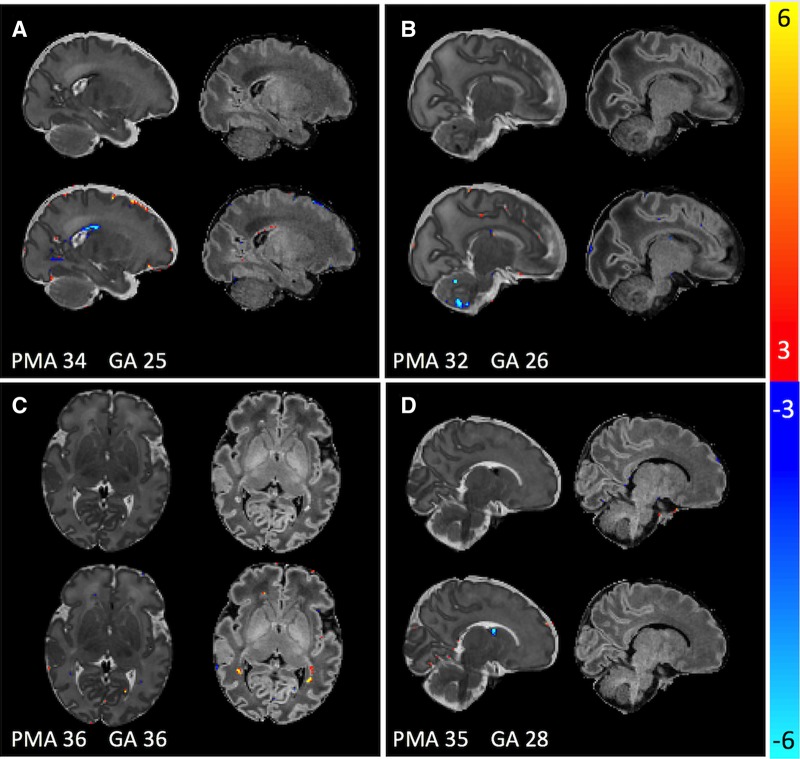
**Four example cases with spatially variable pathologies that are highlighted by the model.** Raw data are in the *top row* of each section, Gaussian process-derived *Z*-scores in the *bottom row*. (**A**) Subject has a posterior germinal matrix haemorrhage, evident as an outlier on T_2_ mainly. (**B**) Subject has several punctate cerebellar haemorrhages, again evident only on T_2_. (**C**) Subject has punctate lesions seen only on T_1_. (**D**) Subject has a germinal matrix haemorrhage in the caudothalamic notch. Pathology visibility using the Gaussian process model reflects the pathological sensitivity of the scan itself. GA = gestational age at birth.

We were able to use these intensity charts to detect and delineate punctate white matter lesions with high accuracy in individual infants. PWMLs are relatively easy to detect visually on T_1_-weighted images (Fig. 5), so they represent a good true positive to evaluate the clinical utility of these models. Therefore, although longitudinal consistency was age-dependent, detection of these tissue abnormalities was not, indicating the possible clinical utility of this type of approach. Use of the GPR model abolished an age-dependence of abnormality detection of PWMLs, providing very high accuracy across the age span (Figs 5 and 6). Although we focused on PWMLs, this sample also included neonates with small or circumscribed haemorrhages, especially in the preterm neonates clearly more evident on T_2_-weighted images, whereas PWML is predominantly evident on T_1_-weighted images (Fig. 7). We did not evaluate regional tissue size in this work, though this is a natural application given our modelling of local and global brain shape.

Even if not using model deviations to quantify abnormality, simply providing estimates of what a ‘normal’ brain should look like alongside clinical MRIs from patients of the same age can improve neuroradiology reporting during this dynamic period (Ou *et al.*, 2017; Prabhu *et al.*, 2018). For this purpose, model parameters can be held and reused to generate new images for a given age and sex as needed by an operator, as well as updated as populations grow or become more diverse.

This study investigated the postnatal brain exclusively. An important future question will be to investigate the relative cross-sectional developmental curves of both the *in* and *ex utero* brain (Bouyssi-Kobar *et al.*, 2016; Lefèvre *et al.*, 2016; Brossard-Racine *et al.*, 2018), allowing a quantitative characterization of how pre- and postnatal brain growth differ at the earliest points in life. Using longitudinal data, it will also be possible to estimate the relationship between foetal and neonatal brain structure in those with prenatally detected brain abnormalities.

A strength of this study is that it used clinical, weighted images. The images were not initially normalized for intensity and the full range of information in the image was used in place of segmenting the scan into a small subset of tissue classes (e.g. grey and white matter probability), as is more typical in brain morphometry. This was straightforward here as all images were of one set of protocols from one scanner (Makropoulos *et al.*, 2018) so the scaling was relatively consistent and comparable across datasets. Using the GPR prediction of the whole image to scale the observed image improved detection of PWMLs. Although this improvement was statistically significant, the effect was small (an increase in AUROC of ∼0.01). Nonetheless, the histograms of the voxelwise deviation scores had a better overlap with normalization (Supplementary Fig. 2). In more variable clinical imaging, image scaling is much more important and some form of adaptation would need to be performed for multi-site studies or studies with different acquisitions. Quantitative MRI techniques, such as diffusion MRI or tissue relaxometry (as in Sadeghi *et al.*, 2013; Dean *et al.*, 2014), would obviate this need for scaling entirely and provide a clearer interpretation of the intensity changes. However, quantitative methods are not in widespread clinical use, limiting the generalizability of such models to real world use, whereas weighted T_1_ and T_2_ contrasts are standard in almost all clinical MRI systems and protocols.

Our models were naïve to the intensities or growth functions in the neighbourhood of the voxels being fit so there was no constraint on the model parameters of surrounding voxels being similar. Though the model can be extended to include neighbourhood information, a simpler approach would be to apply spatial smoothing to the data prior to fitting the model. Explicitly including a patch of voxel intensities as further outputs in the model may have helped further regularize the resulting model parameters, but would come at the cost of increasing the number of parameters in the intrinsic co-regionalization model and may introduce blurring. A reduced-rank approach may be able to address the redundancy in this parameter space, while providing a more global spatial context (Kia and Marquand, 2018).

We also assumed homogeneity of variance across our input variables. From a developmental point of view this is unrealistic, gyrification induces considerably more inter-individual variability as the neonate moves towards term-equivalent age so there is likely more true population variability as infants get older (Dubois *et al.*, 2008). This may be reflected here in the relative blurring of cortex in model predictions of older neonates. A heteroscedastic model (estimating input-dependent noise) or estimating a linear term for the estimated variance may provide a more accurate description of this type of variance. In addition, for model estimation we used a Gaussian likelihood function. This has been shown to be sensitive to outlier values, probable in noisy imaging data and clinically the case here, where spatially heterogeneous incidental findings (as seen in these infants, punctate lesions or small bleeds) could influence model fit and curve shape. In the future, a Student’s *t* process approach, though more computationally expensive, may be more appropriate due to its robustness to outliers (Tracey and Wolpert, 2018).

Perinatal stress, and especially premature birth, has a substantial effect on brain development, conferring later liability to adverse cognitive and health outcomes (Aylward, 2014). Although MRI has shown some sensitivity/specificity for predicting later motor outcomes in those with severe brain injury, there are scant robust links to cognitive and psychiatric outcome (Johnson and Marlow, 2011; van’t Hooft *et al.*, 2015; Batalle *et al.*, 2018). Studies focus on affected groups, rarely individuals, assuming homogeneity in the effect of prematurity that is untenable, and thus likely averaging out effects that are clinically significant on an individual level. Investigating individual neonates as against a growth curve has excellent sensitivity to pathology, uses standard clinical contrast structural MRI and provides an easily interpretable approach to detecting what is atypical in the already atypically developing brain.

## Funding

The developing Human Connectome Project was funded by the European Research Council under the European Union Seventh Framework Programme (FP/20072013), Grant Agreement no. 319456. Infrastructure support was provided by the National Institute for Health Research Mental Health Biomedical Research Centre at South London, Maudsley NHS Foundation Trust, King's College London, the National Institute for Health Research Mental Health Biomedical Research Centre at Guys, and St Thomas’ Hospitals NHS Foundation Trust. The study was supported in part by the Wellcome Engineering and Physical Sciences Research Council Centre for Medical Engineering at King’s College London (grant WT 203148/Z/16/Z) and the Medical Research Council (UK) (grants MR/K006355/1 and MR/LO11530/1). J.O.M. is supported by a Sir Henry Dale Fellowship jointly funded by the Wellcome Trust and the Royal Society (Grant Number 206675/Z/17/Z). J.O.M. and D.E. received support from the Medical Research Council Centre for Neurodevelopmental Disorders, King’s College London (grant MR/N026063/1).

## Competing interests

The authors report no competing interests.

## Supplementary Material

awz412_Supplementary_Materials

## Abbreviations

GPR =: Gaussian process regression
PMA =: post-menstrual age
PWML =: punctate white matter lesions

## References

[awz412-B1] Adler S, Wagstyl K, Gunny R, Ronan L, Carmichael D, Cross JH, et al Novel surface features for automated detection of focal cortical dysplasias in paediatric epilepsy. NeuroImage Clin 2017; 14: 18–27. 28123950 10.1016/j.nicl.2016.12.030 PMC5222951

[awz412-B2] Álvarez MA, Rosasco L, Lawrence ND Kernels for Vector-Valued Functions: A Review [Internet]. ArXiv11066251 Cs Math Stat. 2011 Available from: http://arxiv.org/abs/1106.6251 (30 May 2018, date last accessed).

[awz412-B3] Álvarez MA, Rosasco L, Lawrence ND Kernels for vector-valued functions: a review. Found Trends® Mach Learn 2012; 4: 195–266.

[awz412-B4] Avants BB, Tustison NJ, Song G, Cook PA, Klein A, Gee JC A reproducible evaluation of ANTs similarity metric performance in brain image registration. NeuroImage 2011; 54: 2033–44. 20851191 10.1016/j.neuroimage.2010.09.025 PMC3065962

[awz412-B5] Aylward GP Neurodevelopmental outcomes of infants born prematurely. J Dev Behav Pediatr 2014; 35: 394–407. 25007063 10.1097/01.DBP.0000452240.39511.d4

[awz412-B6] Batalle D, Edwards AD, O’Muircheartaigh J Annual Research Review: not just a small adult brain: understanding later neurodevelopment through imaging the neonatal brain. J Child Psychol Psychiatr 2018; 59: 350–71. 10.1111/jcpp.12838 PMC5900873 29105061

[awz412-B7] Bouyssi-Kobar M, Du Plessis AJ, McCarter R, Brossard-Racine M, Murnick J, Tinkleman L, et al Third trimester brain growth in preterm infants compared with in utero healthy fetuses. Pediatrics 2016; 138: e20161640. 27940782 10.1542/peds.2016-1640 PMC5079081

[awz412-B8] Bozek J, Makropoulos A, Schuh A, Fitzgibbon S, Wright R, Glasser MF, et al Construction of a neonatal cortical surface atlas using Multimodal Surface Matching in the Developing Human Connectome Project. NeuroImage 2018; 179: 11–29. 29890325 10.1016/j.neuroimage.2018.06.018 PMC6783315

[awz412-B9] Brossard-Racine M, McCarter R, Murnick J, Tinkleman L, Vezina G, Limperopoulos C Early extra-uterine exposure alters regional cerebellar growth in infants born preterm. NeuroImage Clin 2018; 21: 101646. 30630759 10.1016/j.nicl.2018.101646 PMC6412008

[awz412-B10] de Bruïne FT, van den Berg-Huysmans AA, Leijser LM, Rijken M, Steggerda SJ, van der Grond J, et al Clinical implications of MR imaging findings in the white matter in very preterm infants: a 2-year follow-up study. Radiology 2011; 261: 899–906. 22031710 10.1148/radiol.11110797

[awz412-B11] Chi JG, Dooling EC, Gilles FH Gyral development of the human brain. Ann Neurol 1977; 1: 86–93. 560818 10.1002/ana.410010109

[awz412-B12] Cordero‐Grande L, Hughes EJ, Hutter J, Price AN, Hajnal JV Three-dimensional motion corrected sensitivity encoding reconstruction for multi-shot multi-slice MRI: application to neonatal brain imaging. Magn Reson Med 2018; 79: 1365–76. 28626962 10.1002/mrm.26796 PMC5811842

[awz412-B13] Dean DC, O’Muircheartaigh J, Dirks H, Waskiewicz N, Walker L, Doernberg E, et al Characterizing longitudinal white matter development during early childhood. Brain Struct Funct 2014; 220: 1921–33. 24710623 10.1007/s00429-014-0763-3 PMC4481335

[awz412-B14] Demšar J Statistical comparisons of classifiers over multiple data sets. J Mach Learn Res 2006; 7: 1–30.

[awz412-B15] Dubois J, Benders M, Cachia A, Lazeyras F, Ha-Vinh Leuchter R, Sizonenko SV, et al Mapping the early cortical folding process in the preterm newborn brain. Cereb Cortex 2008; 18: 1444–54. 17934189 10.1093/cercor/bhm180

[awz412-B16] Dubois J, Germanaud D, Angleys H, Leroy F, Fischer C, Lebenberg J, et al Exploring the successive waves of cortical folding in the developing brain using MRI and spectral analysis of gyrification. In: 2016 IEEE 13th International Symposium on Biomedical Imaging (ISBI). 2016 p. 261–264.

[awz412-B17] Harris JJ, Reynell C, Attwell D The physiology of developmental changes in BOLD functional imaging signals. Dev Cogn Neurosci 2011; 1: 199–216. 22436508 10.1016/j.dcn.2011.04.001 PMC6987565

[awz412-B18] Holland D, Chang L, Ernst TM, Curran M, Buchthal SD, Alicata D, et al Structural growth trajectories and rates of change in the first 3 months of infant brain development. JAMA Neurol 2014; 71: 1266–74. 25111045 10.1001/jamaneurol.2014.1638 PMC4940157

[awz412-B19] van’t Hooft J, van der Lee JH, Opmeer BC, Aarnoudse-Moens CSH, Leenders AGE, Mol BWJ, et al Predicting developmental outcomes in premature infants by term equivalent MRI: systematic review and meta-analysis. Syst Rev 2015; 4: 71. 25982565 10.1186/s13643-015-0058-7 PMC4438620

[awz412-B20] Howell BR, Styner MA, Gao W, Yap P-T, Wang L, Baluyot K, et al The UNC/UMN Baby Connectome Project (BCP): an overview of the study design and protocol development. NeuroImage 2019; 185: 891–905. 29578031 10.1016/j.neuroimage.2018.03.049 PMC6151176

[awz412-B21] Hughes EJ, Winchman T, Padormo F, Teixeira R, Wurie J, Sharma M, et al A dedicated neonatal brain imaging system. Magn Reson Med 2017; 78: 794–804. 27643791 10.1002/mrm.26462 PMC5516134

[awz412-B22] Johnson S, Marlow N Preterm birth and childhood psychiatric disorders. Pediatr Res 2011; 69: 11R–8R. 10.1203/PDR.0b013e318212faa0 21289534

[awz412-B23] Kia SM, Marquand A, Normative modeling of neuroimaging data using scalable multi-task Gaussian processes In: Frangi AF, Schnabel JA, Davatzikos C, Alberola-López C, Fichtinger G, editors. Medical image computing and computer assisted intervention – MICCAI 2018. Springer International Publishing; 2018 p.127–135.

[awz412-B24] Kostović I, Jovanov-Milošević N The development of cerebral connections during the first 20–45 weeks’ gestation. Semin Fetal Neonatal Med 2006; 11: 415–22. 16962836 10.1016/j.siny.2006.07.001

[awz412-B25] Kostović I, Sedmak G, Judaš M Neural histology and neurogenesis of the human fetal and infant brain. NeuroImage 2019; 188: 743–73. 30594683 10.1016/j.neuroimage.2018.12.043

[awz412-B26] Kuklisova-Murgasova M, Aljabar P, Srinivasan L, Counsell SJ, Doria V, Serag A, et al A dynamic 4D probabilistic atlas of the developing brain. NeuroImage 2011; 54: 2750–63. 20969966 10.1016/j.neuroimage.2010.10.019

[awz412-B27] Kuklisova-Murgasova M, Quaghebeur G, Rutherford MA, Hajnal JV, Schnabel JA Reconstruction of fetal brain MRI with intensity matching and complete outlier removal. Med Image Anal 2012; 16: 1550–64. 22939612 10.1016/j.media.2012.07.004 PMC4067058

[awz412-B28] Lebenberg J, Mangin J-F, Thirion B, Poupon C, Hertz-Pannier L, Leroy F, et al Mapping the asynchrony of cortical maturation in the infant brain: a MRI multi-parametric clustering approach. NeuroImage 2019; 185: 641–53. 30017787 10.1016/j.neuroimage.2018.07.022

[awz412-B29] Lefèvre J, Germanaud D, Dubois J, Rousseau F, de Macedo Santos I, Angleys H, et al Are developmental trajectories of cortical folding comparable between cross-sectional datasets of fetuses and preterm newborns? Cereb Cortex 2016; 26: 3023–35. 26045567 10.1093/cercor/bhv123

[awz412-B30] Li G, Wang L, Shi F, Gilmore JH, Lin W, Shen D Construction of 4D high-definition cortical surface atlases of infants: methods and applications. Med Image Anal 2015; 25: 22–36. 25980388 10.1016/j.media.2015.04.005 PMC4540689

[awz412-B31] Liu H, Cai J, Ong Y-S Remarks on multi-output Gaussian process regression. Knowl-Based Syst 2018; 144: 102–21.

[awz412-B32] Lodygensky GA, Thompson DK Toward quantitative MRI analysis: a smart approach to characterize neonatal white matter injury. Neurology 2017; 88: 610–1. 28100729 10.1212/WNL.0000000000003621

[awz412-B33] Mah Y-H, Jager R, Kennard C, Husain M, Nachev P A new method for automated high-dimensional lesion segmentation evaluated in vascular injury and applied to the human occipital lobe. Cortex J Devoted Study Nerv Syst Behav 2014; 56: 51–63. 10.1016/j.cortex.2012.12.008 PMC4071441 23347558

[awz412-B34] Makropoulos A, Aljabar P, Wright R, Hüning B, Merchant N, Arichi T, et al Regional growth and atlasing of the developing human brain. NeuroImage 2016; 125: 456–78. 26499811 10.1016/j.neuroimage.2015.10.047 PMC4692521

[awz412-B35] Makropoulos A, Robinson EC, Schuh A, Wright R, Fitzgibbon S, Bozek J, et al The developing human connectome project: a minimal processing pipeline for neonatal cortical surface reconstruction. NeuroImage 2018; 173: 88–112. 29409960 10.1101/125526 PMC6783314

[awz412-B36] Marquand AF, Rezek I, Buitelaar J, Beckmann CF Understanding heterogeneity in clinical cohorts using normative models: beyond case-control studies. Biol Psychiatry 2016; 80: 552–61. 26927419 10.1016/j.biopsych.2015.12.023 PMC5023321

[awz412-B37] McCarty DB, Peat JR, Malcolm WF, Smith PB, Fisher K, Goldstein RF Dolichocephaly in preterm infants: prevalence, risk factors, and early motor outcomes. Am J Perinatol 2017; 34: 372–8. 27588933 10.1055/s-0036-1592128

[awz412-B38] Mewes AUJ, Zöllei L, Hüppi PS, Als H, McAnulty GB, Inder TE, et al Displacement of brain regions in preterm infants with non-synostotic dolichocephaly investigated by MRI. NeuroImage 2007; 36: 1074–85. 17513129 10.1016/j.neuroimage.2007.04.011 PMC3358776

[awz412-B39] Mills KL, Tamnes CK Methods and considerations for longitudinal structural brain imaging analysis across development. Dev Cogn Neurosci 2014; 9: 172–90. 24879112 10.1016/j.dcn.2014.04.004 PMC6989768

[awz412-B40] Morel B, Antoni G, Teglas JP, Bloch I, Adamsbaum C Neonatal brain MRI: how reliable is the radiologist’s eye? Neuroradiology 2016; 58: 189–93. 26494461 10.1007/s00234-015-1609-2

[awz412-B41] Oishi K, Chang L, Huang H Baby brain atlases. NeuroImage 2019; 185: 865–80. 29625234 10.1016/j.neuroimage.2018.04.003 PMC6170732

[awz412-B42] O’Muircheartaigh J, Dean DC, Dirks H, Waskiewicz N, Lehman K, Jerskey BA, et al Interactions between white matter asymmetry and language during neurodevelopment. J Neurosci 2013; 33: 16170–7. 24107949 10.1523/JNEUROSCI.1463-13.2013 PMC3792458

[awz412-B43] O’Muircheartaigh J, Vavasour I, Ljungberg E, Li DKB, Rauscher A, Levesque V, et al Quantitative neuroimaging measures of myelin in the healthy brain and in multiple sclerosis. Hum Brain Mapp 2019; 40: 2104–16. 30648315 10.1002/hbm.24510 PMC6590140

[awz412-B44] Ou Y, Zöllei L, Retzepi K, Castro V, Bates SV, Pieper S, et al Using clinically-acquired MRI to construct age-specific ADC atlases: quantifying spatiotemporal ADC changes from birth to 6 years old. Hum Brain Mapp 2017; 38: 3052–68. 28371107 10.1002/hbm.23573 PMC5426959

[awz412-B45] Peterson BS, Anderson AW, Ehrenkranz R, Staib LH, Tageldin M, Colson E, et al Regional brain volumes and their later neurodevelopmental correlates in term and preterm infants. Pediatrics 2003; 111: 939–48. 12728069 10.1542/peds.111.5.939

[awz412-B245] Prabhu SP, Gatowski A, Kalafut J, Johnson P, Robertson RL Effect of contextual age-matched normative reference images on pediatric brain MRI for white matter disease. Maryland: Proceedings of the Society of Imaging Informatics in Medicine (SIIM); 2018.

[awz412-B46] Rasmussen CE, Williams C Gaussian processes for machine learning, Vol. 38 Cambridge, MA, USA: MIT Press; 2006 p. 715–719.

[awz412-B47] Robinson EC, Jbabdi S, Glasser MF, Andersson J, Burgess GC, Harms MP, et al MSM: a new flexible framework for Multimodal Surface Matching. NeuroImage 2014; 100: 414–26. 24939340 10.1016/j.neuroimage.2014.05.069 PMC4190319

[awz412-B48] Rutherford M, Srinivasan L, Dyet L, Ward P, Allsop J, Counsell S, et al Magnetic resonance imaging in perinatal brain injury: clinical presentation, lesions and outcome. Pediatr Radiol 2006; 36: 582–92. 16770663 10.1007/s00247-006-0164-8

[awz412-B49] Sadeghi N, Prastawa M, Fletcher PT, Wolff J, Gilmore JH, Gerig G Regional characterization of longitudinal DT-MRI to study white matter maturation of the early developing brain. NeuroImage 2013; 68: 236–47. 23235270 10.1016/j.neuroimage.2012.11.040 PMC3693970

[awz412-B50] Salmond CH, Ashburner J, Vargha-Khadem F, Connelly A, Gadian DG, Friston KJ Distributional Assumptions in Voxel-Based Morphometry. NeuroImage 2002; 17: 1027–30. 12377176

[awz412-B51] Sled JG, Nossin-Manor R Quantitative MRI for studying neonatal brain development. Neuroradiology 2013; 55: 97–104. 23872867 10.1007/s00234-013-1235-9

[awz412-B52] Smith SM, Jenkinson M, Woolrich MW, Beckmann CF, Behrens TEJ, Johansen-Berg H, et al Advances in functional and structural MR image analysis and implementation as FSL. NeuroImage 2004; 23 (Suppl 1): S208–219. 15501092 10.1016/j.neuroimage.2004.07.051

[awz412-B53] Tracey BD, Wolpert DH Upgrading from Gaussian Processes to Student’s-T Processes [Internet]. 2018 Available from: https://arxiv.org/abs/1801.06147v1 (3 May 2019, date last accessed).

[awz412-B54] Van Essen DC A Population-Average, Landmark- and Surface-based (PALS) atlas of human cerebral cortex. NeuroImage 2005; 28: 635–62. 16172003 10.1016/j.neuroimage.2005.06.058

[awz412-B55] Volpe JJ Brain injury in premature infants: a complex amalgam of destructive and developmental disturbances. Lancet Neurol 2009; 8: 110–24. 19081519 10.1016/S1474-4422(08)70294-1 PMC2707149

[awz412-B56] Ziegler G, Ridgway GR, Dahnke R, Gaser C Individualized Gaussian process-based prediction and detection of local and global gray matter abnormalities in elderly subjects. Neuroimage 2014; 97: 333–48. 24742919 10.1016/j.neuroimage.2014.04.018 PMC4077633

