# Corrigendum

**DOI:** 10.1093/brain/awaa048

**Published:** 2020-02-21

Jonathan O’Muircheartaigh, Emma C. Robinson, Maximillian Pietsch, Thomas Wolfers, Paul Aljabar, Lucilio Cordero Grande, Rui P.A.G. Teixeira, Jelena Bozek, Andreas Schuh, Antonios Makropoulos, Dafnis Batalle, Jana Hutter, Katy Vecchiato, Johannes K. Steinweg, Sean Fitzgibbon, Emer Hughes, Anthony N. Price, Andre Marquand, Daniel Reuckert, Mary Rutherford, Joseph V. Hajnal, Serena J. Counsell, A. David Edwards. Modelling brain development to detect white matter injury in term and preterm born neonates. Brain 2020; 143: 467–479. doi:10.1093/brain/awz412.

The authors apologize for misspelling the name of the authors Emma C. Robinson, Joseph V. Hajnal, Anthony N. Price and A. David Edwards. These have now been corrected.

