# Supplementary material for: Modelling brain development to detect white matter injury in term and preterm born neonates
Source: Brain. 2020 Jan 16;143(2):467–79. doi: 10.1093/brain/awz412 (PMC7009541; doi:10.1093/brain/awz412)
Supplement: awz412_Supplementary_Materials [file awz412_supplementary_materials.zip › awz412-suppl_data/brain-2019-01281-File010.pdf]

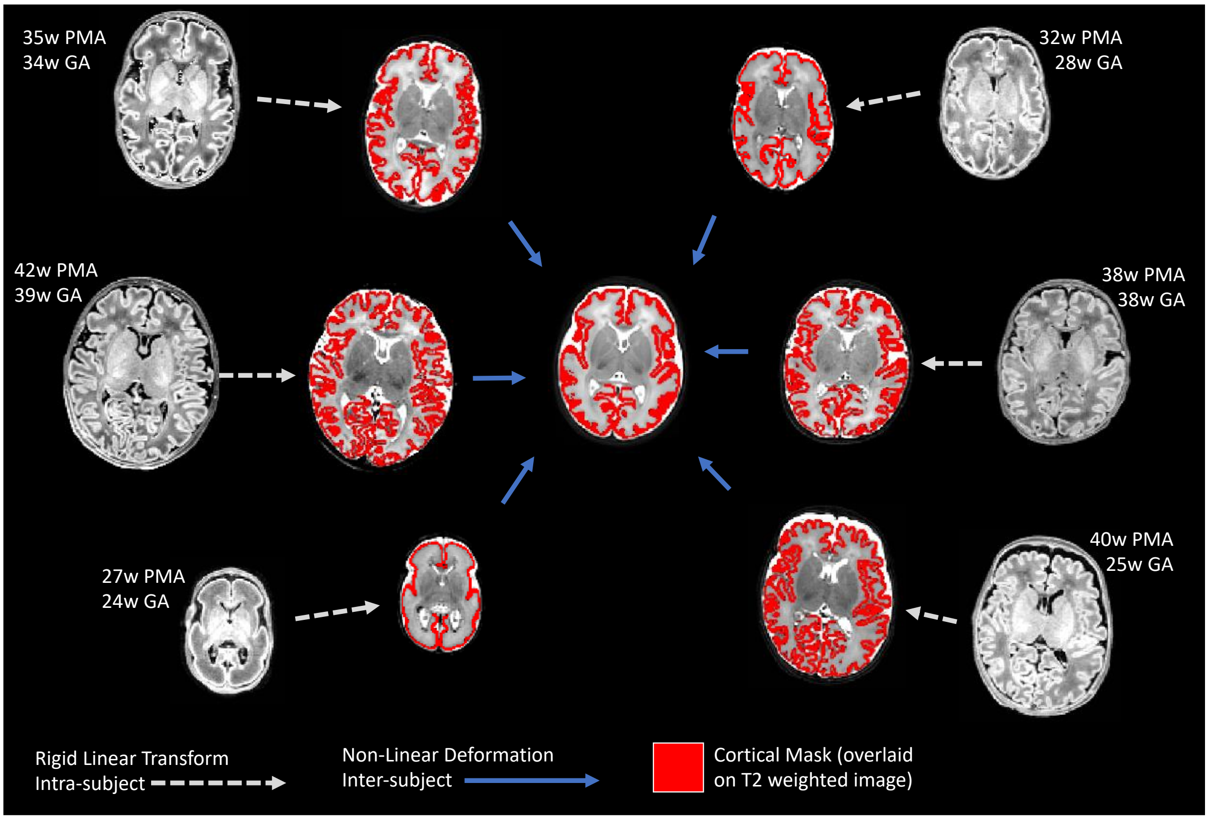

Supplementary Figure 1: An illustration of the image registration pipeline. All individual scans are registered to the study specific template using non-linear registration using a combination of two features: the T2 weighted image intensity and the estimated cortex. T1 weighted images are registered to the T2 weighted scan using rigid (linear) alignment. Both T1 and T2 weighted images are resampled in template space in a single step. The resulting template (middle) is representative of roughly 37 weeks gestational age.

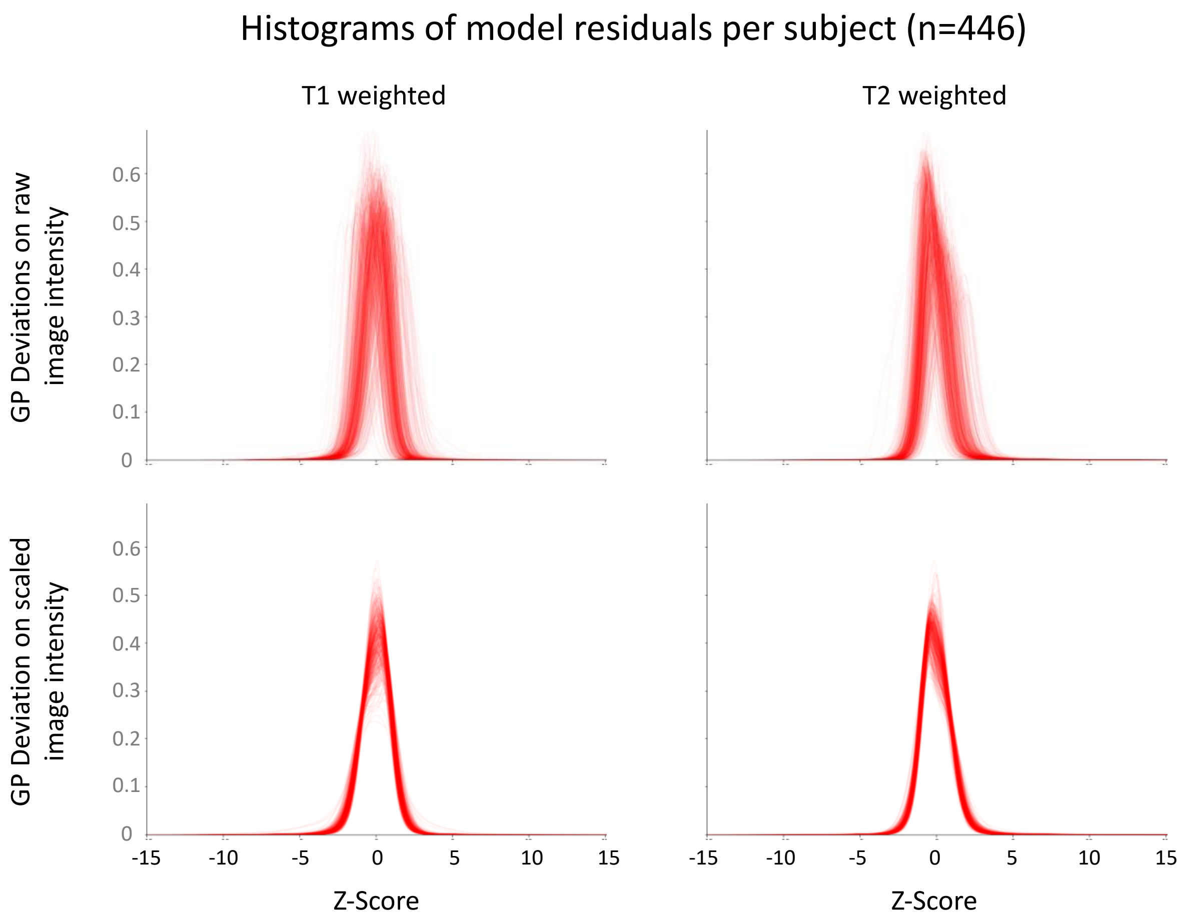

Supplementary Figure 2: Histograms of the residual distribution of deviation scores for the T1 and T2 weighted images of all the neonates after cross validation. Intensity normalisation by the median of the GP predicted intensity across each scan provided a clearer overlap of residual distributions across subjects.

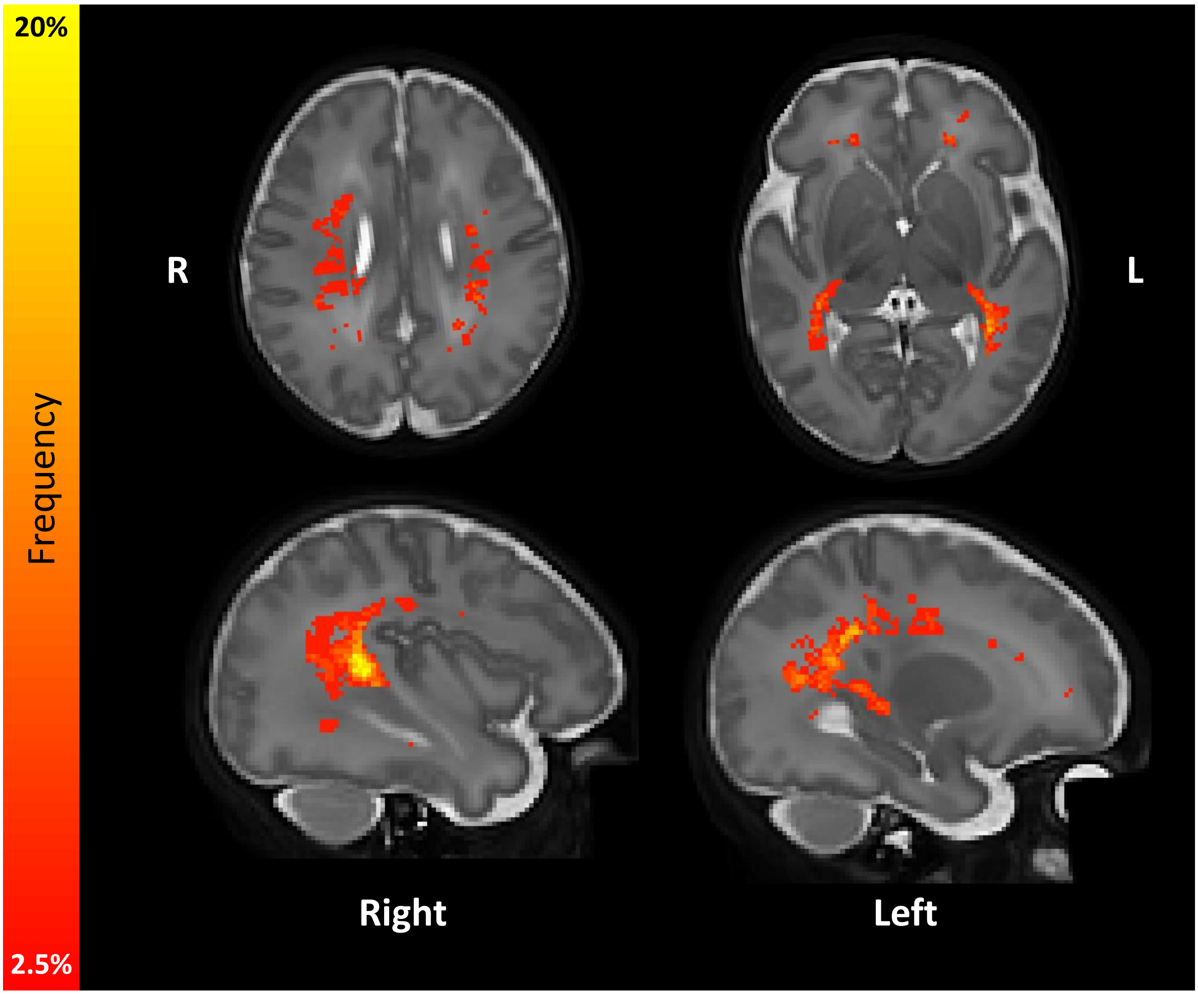

Supplementary Figure 3: Punctate white matter lesion maps illustrated as a frequency map on the T2 weighted template image.
